# Barriers to coliphage infection of commensal intestinal flora of laboratory mice

**DOI:** 10.1186/1743-422X-2-34

**Published:** 2005-04-15

**Authors:** Laura M Kasman

**Affiliations:** 1 Department of Microbiology and Immunology, Medical University of South Carolina, BSB-201, P.O. Box 250504, 173 Ashley Avenue, Charleston, SC 29403, USA

## Abstract

**Background:**

Growth characteristics of coliphage viruses indicate that they are adapted to live with their *Eschericia coli *hosts in the intestinal tract. However, coliphage experimentally introduced by ingestion persist only transiently if at all in the gut of humans and other animals. This study attempted to identify the barriers to long term establishment of exogenous coliphage in the gastrointestinal (GI) tracts of laboratory mice. Intestinal contents were screened for the presence of coliphage and host bacteria, and strains of *E. coli *bacteria from different segments of the GI tract were tested for susceptibility to six common laboratory coliphages.

**Results:**

Contrary to expectations, coliphage were not evident in the GI tracts of laboratory mice, although they were occasionally detected in feces. Commensal flora showed extreme variability within groups of mice despite identical handling and diet. Less than 20% of 48 mice tested carried *E. coli *in their gut, and of 22 commensal *E. coli *strains isolated and tested, 59% were completely resistant to infection by lambda, M13, P1, T4, T7, and PhiX174 coliphage. Lysogeny could not be demonstrated in the commensal strains as mitomycin C failed to induce detectable phage. Pre-existing immunity to phages was not evident as sera and fecal washes did not contain significant antibody titers to six laboratory phage types.

**Conclusion:**

Lack of sufficient susceptible host bacteria seems to be the most likely barrier to establishment of new coliphage infections in the mouse gut.

## Background

Coliphage have traditionally been isolated from sewage, where they arrived, presumably, after passing through the GI tracts of animals inhabited by commensal coliform bacteria. However, information on the interaction of natural coliphage with the commensal flora of the GI tract in situ is sparse. The results of ingestion of defined high titer bacteriophage preparations by laboratory animals or by humans have been described in many previous studies (reviewed in [1]). Although there is evidence in some reports of coliphage replication in the gut, the phage infections are consistently transient, becoming undetectable in 3–10 days [2-4]. Notable exceptions are cases of gnotobiotic mice inoculated with defined phage-host systems, in which phage and host populations in feces were detectable for up to 98 days [4]. However, in animals with complex, established gastrointestinal microflora, observations concur that exogenous phage do not establish sustained productive infections of the commensal bacteria. The nature of this apparent barrier to persistent bacteriophage infection of the normal GI tract is of practical interest since as parasites of commensal bacteria, bacteriophage have the potential to impact health by altering the GI flora. Conversely, it may be possible to engineer phage specifically to alter the commensal flora in situ for therapeutic benefit.

Previous work has already shown that potential physical obstacles to bacteriophage infection of bacteria in the gut are not significant. The acid environment of the stomach would at first appear to be an obvious barrier, but coliphage have been shown to maintain their infectivity when passed through the stomach or exposed to gastric fluids [4-7]. Attachment of phage to the host bacteria could be inhibited by secretions or deficiencies of cofactors in the GI environment, however infection of bacteria in the gut has also been demonstrated for a variety of phage [5,8,9], including the temperate phage, CTXphi, of *Vibrio cholerae*. CTXphi in fact infects its host more efficiently in vivo than in vitro, because the phage has adapted to use as its receptor *V. cholerae *surface molecules expressed only when the bacteria colonizes the gut [10]. However, *V. cholerae *is not normal flora, and CTXphi lysogens and phage are cleared in the usual time frame. In addition, antimicrobial phage therapy trials have demonstrated phage infection of bacteria in the peritoneal cavity, blood, muscle [1,11] and embryonated hen eggs (L. Kasman, unpublished observations). Therefore, the in vivo environment itself does not directly prevent infection of bacteria by phage.

Phage are immunogenic, even when introduced into the GI tract [5,9] and so anti-phage antibodies may play a role in the barrier to new phage infections in commensal coliforms. Alternatively, susceptible host bacteria may not be present at high enough densities to allow persistent infection by coliphage. When phage concentration are low, very high densities of host cells can be required to enable a detectable number of infections to occur[12]. Commensal intestinal bacteria are a climax community in which an estimated 400 different species of bacteria fill all niches with regard to locations and energy sources, so that newcomers find it difficult or impossible to become established [13,14]. A new bacteriophage could in theory face a similar obstacle, although not in terms of metabolic needs, as most phage lysogens inhibit superinfection of their host by similar phage types.

In this study, these potential barriers phage colonization of commensal bacteria were assessed for *E. coli*, by testing the gut contents and feces of several strains of laboratory mice for the existence of free coliphage and *E. coli *capable of supporting phage infection. In addition, six well characterized coliphage types were used to test commensal *E coli *bacteria for susceptibility to phage infection, and sera and mucosal secretions were examined for evidence of anti-coliphage antibodies.

## Results

### Screening for endogenous coliphage in the feces of laboratory mice

Feces of two independent groups of 40 and 48 ICR mice, were screened every three days for a total of four samplings for the presence of naturally occurring (endogenous) coliphage. Fresh fecal pellets were collected for each mouse individually, resuspended in LB, then centrifuged and the supernatants spotted onto indicator *E. coli *in top agar within 6 h of collection. Feces of small groups of C57BL/6 and nude mice were also collected such that over 400 samples were screened on indicator *E. coli *strains CN13 (F-) and ER2738 (F+) for somatic and male-specific phages respectively. A total of three samples from two mice were positive for phage on the somatic phage host only. All other feces samples were negative, indicating that even in the mice positive for phage, the phage could only be detected transiently.

### Screening for coliphage and lysogens in the murine intestinal tract

In order to investigate coliphage prevalence in the GI tract as well as feces, 48 ICR and 15 C57BL/6 mice were euthanized after a final feces collection, and GI tracts removed aseptically to individual sterile petri dishes. Each GI tract was divided into four segments: (1) stomach and duodenum, (2) 10 cm of small intestine midway between the duodenum and cecum, (3) cecum, and (4) colon and rectum. Intestinal contents were collected by flushing each segment with 1 ml L-Broth containing CaCl_2 _and MgSO_4_, into a microfuge tube. Samples were gently vortexed, subjected to low speed centrifugation to pellet large particulates, and the supernatants retained for phage detection as described below. Known amounts of T4 and M13 phage were added to rinses of one mouse as a control, which demonstrated that the collection procedure and centrifugation did not remove or destroy phage (data not shown).

Due to the near absence of detectable phage in ICR mouse feces initially collected, two strategies to enhance coliphage detection were applied to the supernatants from the murine GI tracts and final feces collection from ICR, C57BL/6 and nude mice. First, in order to amplify phage which might be present at levels too low to detect, permissive *E. coli *host strains ER2738 or CN-13 were added to liquid cultures containing aliquots of gut or fecal supernatants. Second, mitomycin C was added since coliphage lysogens, which are otherwise undetectable by plaque assay, can usually be reactivated to the free state by treatment of the bacteria with the chemical mitomycin C. Combinations of permissive cells and mitomycin C produced five different culture conditions for the enrichment of phage recovery from feces and gut contents (Table 1). Supernatants from overnight cultures under these conditions were assayed for the presence of phage by spotting on ER2738 and CN13 agar overlays. Table 1 summarizes the results of several experiments. No male-specific bacteriophage were detected in gut contents or feces of any laboratory mice from two suppliers and three animal strains (nude mice were analyzed by feces only). Somatic bacteriophage were detected in the feces but not the gut contents of one ICR mouse after enrichment, regardless of mitomycin C treatment. Mitomycin C failed to produce any more detectable coliphage.

**Table 1:** Titers of bacteriophage after enrichment cultures from murine feces and contents of the GI tract

		Enrichment method				
		
		1	2	3	4	5
Mouse strain	source	No additions	CN-13 (F-) bacteria	CN-13 (F-) bacteria With mitomycin C	ER2738(F+) bacteria	ER2738(F+) bacteria With mitomycin C

ICR	feces	-^2^	7 × 10^5 ^PFU^3^	6 × 10^5 ^PFU^3^	-	-
C57BL/6	feces	-	-	-	-	-
BALB/c nude	feces	-	-	-	-	-
ICR	GI^1^	-	-	-	-	-
C57BL/6	GI^1^	-	-	-	-	-

^1 ^All four compartments were tested separately

^2 ^– represents none detected. Minimum detectable titer was 10^2^/ml before enrichment.

^3 ^Average of two mice, out of 88 tested. All others were negative.

### Prevalence of commensal *E. coli *in laboratory mice

Prevalence of commensal *E. coli *was studied in 48 six-week old female ICR mice, shipped together and housed in 12 groups of 4 mice for two weeks in the MUSC animal facility, maintained on a diet of tap water and sterilized pellets. Aliquots of fecal suspensions and intestinal segment rinses for all mice were plated on individual MaConkey agar plates. Although large numbers of bacteria grew from all of the samples except some duodenum segments, less than half of the plates had colonies with morphology and color consistent with *E. coli*. Sixty-four individual colonies were chosen from 64 different plates and typed using the BioMerieux api20E system. It was found that only 20 of the 64 selected colonies were *E. coli*, 36 were *Klebsiella teragina *or *Enterobacter aerogenes*, 3 were *Klebsiella ornithinolytica*, 1 was *Klebsiella pneumoniae pneumoniae*, 1 was *Myroides chryseobacterium indologenes*, and 3 could not be identified. The 20 *E. coli *isolates were from 8 mice, so that only 8 of 48, or 17%, of the mice were found to carry *E. coli. E. coli *prevalence and diversity of commensal flora was similar in subsequent smaller groups of mice surveyed.

### Susceptibility of commensal *E. coli *to laboratory-adapted coliphage

In total, 31 commensal *E. coli *strains recovered from four different gastrointestinal compartments and verified by BioMerieux species identification were collected from both ICR and C57BL/6 mice. These strains were tested for susceptibility to six well-characterized laboratory adapted coliphage (Lambda, M13, P1, øX174, T4, and T7) by spotting of two dilutions of each phage onto an agar overlay inoculated with the strain to be tested. Divalent cations CaCl_2 _and MgSO_4 _were supplied in the overlay as they are required for infection by P1. Susceptibility was observed as confluent lysis or individual plaques in the bacterial lawn after 18 h incubation. Isolates from different compartments of the same mouse that had identical phage susceptibility profiles were considered to be the same strain, reducing the number of unique isolates from 31 to 22. Less than 50% of these commensal strains were susceptible to any of the six coliphage (Table 2). None of the 22 strains were susceptible to M13mp18 or lambda phage. P1 was the broadest host range phage, and was able to lyse 8 of 22 strains (36%) although plaques were considerably smaller in all cases compared to T4, T7 or øX174. Similar resistance was found in a panel of 15 human clinical *E. coli *isolates tested for susceptibility in the same manner (Table 2).

**Table 2:** Susceptibility of commensal *E. coli *strains to lysis by laboratory coliphage (percent susceptible strains)

**Source of *E. coli***	**Lambda**	**M13**	**P1**	**øX174**	**T4**	**T7**	**none**
Mouse feces and GI tracts (n = 22)	0 (0%)	0 (0%)	8 (36%)	3 (14%)	4 (18%)	3 (14%)	13 (59%)
Human clinical specimens (n=15)	1 (7%)	1 (7%)	6 (40%)	1 (7%)	1 (7%)	2 (13%)	7 (47%)

### Screening of mouse serum and mucosal secretions for anti-coliphage antibodies

**Figure 1:**
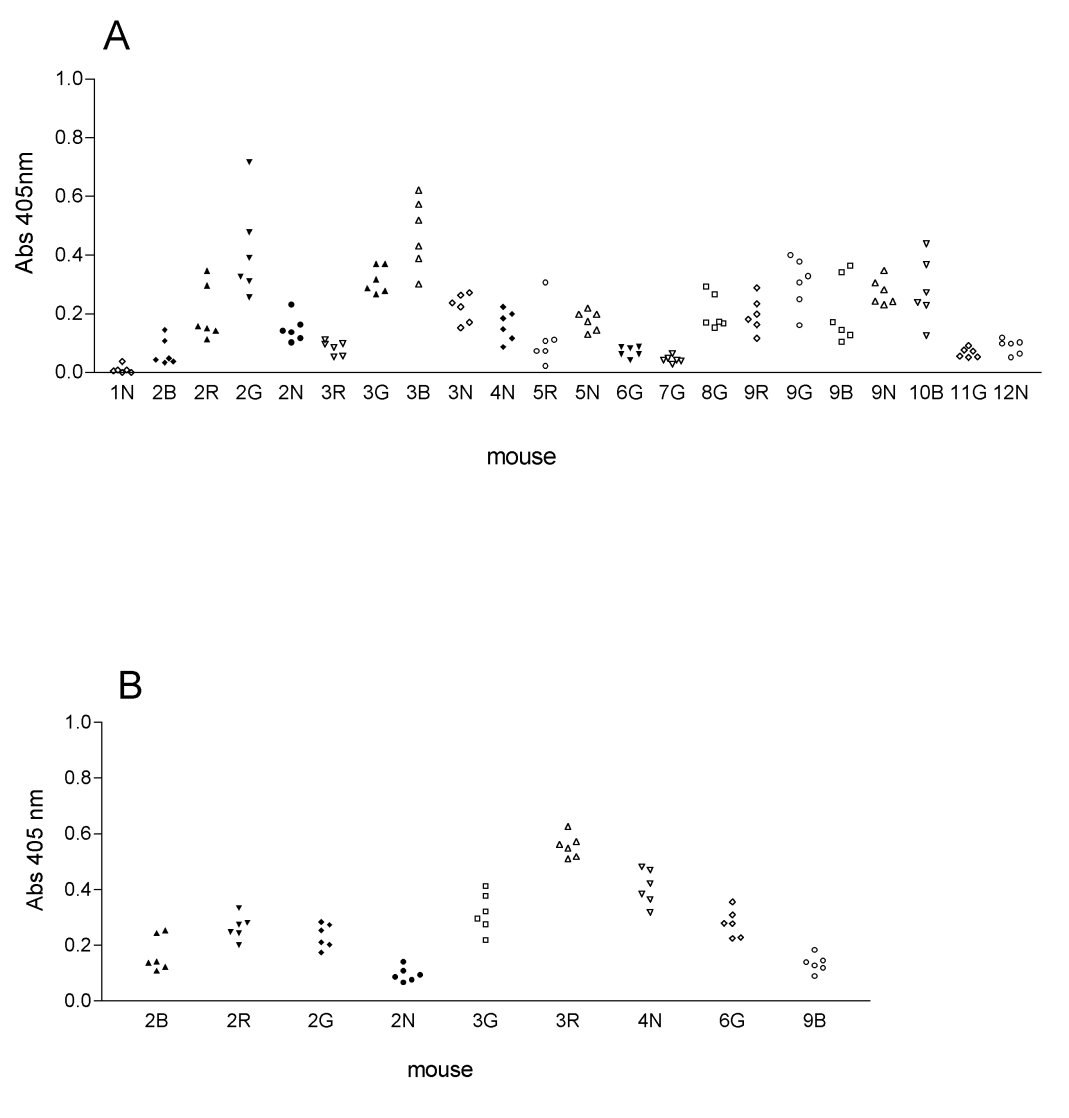
**Absence of specific antibodies to commensal coliphage by ELISA**. (A) Fecal washes from 22 ICR mice found to be colonized with lactose fermenting commensal bacteria were assayed in duplicate for IgA antibodies against M13mp18, Lambda, P1, øX174, T4 and T7 coliphages in parallel ELISA plates. Mice 2B, 2R, 2G, 2N, 3G, 3R, 4N, and 6G were eventually shown to carry *E. coli*. Each symbol represents the mean absorbance reading for one phage type. (B) Sera from 9 mice verified as *E. coli *carriers were assayed in duplicate for IgG and IgM antibodies against the same six coliphage by ELISA. Each symbol represents the mean absorbance reading for one phage type.

Pre-existing immunity in the form of mucosal or serum antibodies might account for the absence of coliphage in the gut. Fecal washes from 22 mice positive for lactose fermenting commensals were screened for IgA antibodies against M13, Lambda, P1, PhiX174, T4 or T7 phages by ELISA. There was variability in IgA reactivity between mice, but each individual mouse had similar reactivity to all 6 phages (Figure 1A). Based on a cut-off of two standard deviations from the mean IgA response for any given mouse, there was no evidence of phage specific mucosal IgA antibodies in this group of animals. Sera from 9 mice verified to be carriers of *E. coli *were likewise assayed for the presence of IgG and IgM antibodies. No evidence of phage-specific serum antibodies were found in any of the mice (Fig 1B)

## Discussion

Bacteriophages are assumed to be a normal component of mammalian gastrointestinal microbial flora since they are commonly isolated from feces and raw sewage and grow most efficiently at temperatures approximating mammalian body temperature. This study focused on the bacteriophages of E. coli, since it is the commensal bacterial species for which the most characterized phages are known and it is easily grown in vitro. We also primarily used outbred mice (strain ICR) to more closely approximate a human population, although diet and environment were kept homogeneous. Contrary to expectations, with the exception of transient somatic phage infections in the feces of 2 of 88 mice, coliphage were not evident in the GI tracts of laboratory mice examined in this study. Our detection limit was 100 PFU/ml for GI tract rinses and approximately 100 PFU/gram feces before enrichment. However, in theory, a single infectious phage particle or single lysogenic cell should be detectable after enrichment. Coliphage densities in feces of domestic animals have been reported to vary widely by species [15]. Our results suggest that laboratory mice have some of the lowest fecal phage densities tested for domestic animals, but comparable to that reported for humans [15]. This was true for mice from two suppliers, and could have been a result of intentional association of the laboratory animals with a defined flora at the breeding facility. However, a defined flora also was not evident in these animals. At least with regard to aerobes, the commensal flora of the mice was very diverse despite the fact that the animals were all maintained in a barrier facility and fed the identical sterilized chow and water. *E. coli*, in particular, were detected in less than 20% of the animals, and 54% of animals produced no lactose fermenters at all on MaConkey agar.

The finding that murine commensal *E. coli *strains isolated were mostly resistant to infection by lambda, M13, P1, T4, T7, and PhiX174 coliphage was at first suggestive of a climax community of phage. However, superinfection exclusion became an unlikely mechanism for this resistance given the lack of phage production in response to mitomycin C. In addition, no phage-specific antibodies to known coliphage types were found in the sera or fecal washes of mice known to be colonized with *E. coli*. Phage are well known to be highly immunogenic in mice when injected and to pass into the blood when ingested in large numbers [7]. Taken together, these results indicate that the inability to establish persistent coliphage infections in commensal flora was not due to a climax community of phage already existing in the mouse.

Given the small percentage of commensal *E. coli *strains found to be susceptible to known coliphage types, the main barrier to establishment of new coliphage infections in the gut was most likely insufficient host cell density. It is estimated that in human *E. coli *carriers, 1 in 10,000 bacterial cells in the gut is an *E. coli *cell, and one gram of normal human feces contains roughly 10^6 ^live *E. coli*. If ideal conditions were present, such that there were 10^6 ^susceptible cells per ml of gut content, and the gut is assumed to approximate a liquid environment, then it can be calculated that a given phage present at a concentration of only 10 PFU/ml would be expected to infect on average two *E. coli *cells per ml of gut per hour[12]. However, conversely, in order to infect every available *E. coli *cell in such ideal conditions would require a phage concentration exceeding 1.5 × 10^6 ^PFU/ml. Based on our experience, the mouse intestinal volume is less than 3 mls, and the susceptible *E. coli *population is much lower. In addition, the majority of intestinal bacteria are located in the cecum and colon, where they will be eliminated from the body the most rapidly. It should be noted that the majority of microorganisms in the mammalian gut are anaerobes. Very little is known about bacteriophage of these organisms, but is possible that bacteriophages of anaerobes or bacteria other than *E. coli *would produce a different outcome.

## Conclusion

The purpose of this investigation was to identify the barriers to long term establishment of coliphage in the GI tracts of laboratory mice. It was found that (1) laboratory animals living under identical conditions and with an identical diet can have very different populations of commensal intestinal bacteria, (2) *E. coli*, while common in human intestinal tracts were relatively rare in the guts of laboratory mice, (3) commensal *E. coli *found in the GI tract of normal laboratory mice were resistant to several types of coliphage. In conclusion, lack of sufficient susceptible host bacteria seems to be the most likely barrier to establishment of new coliphage infections in the mammalian gut. The results suggest that altering commensal *E. coli *with coliphage in situ will be very difficult due to the high variability of commensal flora between individuals, and the low probability of a coliphage particle making contact with a susceptible cell in the gut.

## Methods

### Bacterial strains and coliphage

Male specific phage host strain *E. coli *ER2738 (F'proA^+^B^+ ^lacI^q ^delta(lacZ)M15zzf::Tn10(Tet^R^) and M13mp18 coliphage were obtained from New England Biolabs (Beverly MA). Somatic phage host *E. coli *strain CN13, and coliphages T4 and T7 were purchased from the American Type Culture Collection, Rockland MD. Coliphage P1 and PhiX174 were from generously provided by Dr. Caroline Westwater (Medical University of South Carolina). Lambda gt11 (Promega, Madison, WI). was generously provided by Phillip Werner, Medical University of South Carolina. *E. coli *bacterial strains grown from de-identified human clinical specimens were provided by Dr. Lisa Steed, Dept. of Pathology and Laboratory Medicine, MUSC. Bacteria were grown in Luria Broth with or without agar (Difco) or on Maconkey agar (Difco) as noted. Mitomycin C was purchased from Sigma and used at a concentration of 2 μg/ml.

### Mice

Female ICR and C57BL/6 mice were obtained from Harlan Laboratories, C57BL/6 mice were also obtained from Jackson Laboratories. Feces from nude mice were provided by Dr. Mark Hyer (Medical University of South Carolina). All mice were housed and cared for in the MUSC animal facility and handled according to protocols approved by the MUSC Institutional Animal Care and Use Committee.

### Survey for lysogeny and isolation of *E. Coli *strains

Gastrointestinal tracts from adult ICR and C57/BL6 mice were collected from fresh cadavers and divided into four segments: (1) stomach and duodenum, (2) 10 cm of small intestine midway between the duodenum and cecum, (3) cecum, and (4) colon and rectum. Intestinal contents were collected by flushing each segment with 1 ml L-Broth containing 5 mM CaCl_2 _and 10 mM MgSO_4_, vortexed briefly, and centrifuged at 50 RCF to remove large particulates. Aliquots of supernatant were plated onto MaConkey agar to isolate commensal *E. coli*. Remaining supernatant was divided and incubated under 5 different conditions to enrich for coliphage: (1) No added host bacteria, no mitomycin C, (2) CN-13 host bacteria and 2 μg mitomycin C/ml, (3) CN-13 host bacteria and no mitomycin C, (4) ER2738 host bacteria and 2 μg mitomycin C/ml, (5) ER2738 host bacteria and no mitomycin C. After 18 h shaking at 37°C, enriched cultures were pelleted by centrifugation at 20,800 RCF. The presence of male-specific phage and somatic phage in the supernatant was assayed by spotting 10 μl onto host cells according to Method 1601 of the United States Environmental Protection Agency for detection of coliphage in fresh water samples[16], except that New England Biolabs strain ER2738 was substituted for strain F_amp _*E. coli*. Fecal washes were prepared by resuspending fecal pellets in PBS containing 0.2% sodium azide at a ratio of 100 mg feces per ml. Particulates were pelleted at 14 krpm in a microfuge and the supernatants frozen until analysis.

### Spotting Assay for presence of phage and analysis of phage susceptibility

The EPA protocol for detection of coliphage in drinking water[16] was followed except that *E. coli *strain ER2738 was substituted for strain F_amp_. Fecal pellets were resuspended as a 10% slurry (weight to volume) in LB containing 10 mM CaCl_2 _and MgSO_4_. For detection of phage in feces, slurries were centrifuged 10 minutes at 14 Krpm in a microcentrifuge to remove particulates before spotting 10 μl onto *E. coli *inoculated top agar overlay. For determination of phage susceptibility, verified commensal *E. coli *strains or ER2738 and CN13 control strains were grown to logarithmic phase in LB and 0.1 ml of culture used to inoculate 3 mls molten LB top agar (0.6% agar, 42°C) which was poured onto an LB agar base. When top agars were dry, 10 μl intestinal wash or 5 μl of two dilutions of lab adapted phage (Lambda, M13mp18, P1, PhiX174, T4, and T7) were spotted onto the top agar. For phage susceptibility testing, the highest dilution producing complete clearing on the control strain, and the dilution ten-fold more concentrated were used. Plates were read after incubation 18–20 h at 37°C.

### Bacterial species verification

For isolation and verification of commensal *E. coli *strains, single bacterial colonies with suspected *E. coli *morphology were picked from MaConkey agar plates inoculated with gut contents or feces. Each colony was restreaked on a separate MaConkey agar plates and single colonies analyzed with the BioMerieux api20e typing kit for species identification according to manufacturer's instructions (BioMerieux, Inc. Durham, NC).

### ELISA

ELISAs were performed in Reacti-Bind™ maleic anhydride activated clear 96-well plates (Pierce, Rockford IL) coated overnight with one of 6 phages (M13mp18, Lambda, P1, øX174, T4 or T7) in sterile filtered bacterial culture supernatants diluted in PBS. Fecal washes were tested for the presence of phage-specific IgA antibodies, at a dilution of 1:3 in duplicate wells for 2 h at room temperature, using a goat anti-mouse IgA-alkaline phosphatase conjugated secondary antibody (Southern Biotechnology Assoc., Birmingham AL) and Super Signal chemiluminescent substrate (Pierce). Mouse sera were incubated in the same manner except at a 1:6 dilution and utilizing secondary HRP-conjugated antibodies recognizing mouse IgG or IgM (Sigma) to detect phage-specific antibodies. Sera from mice immunized with the laboratory phages by intraperitoneal injection served as positive controls. Horseradish peroxidase conjugated secondary antibodies were detected by incubation with 1-Step ABTS (Pierce).

## Competing interests

The author received salary and support from September 1999 to May 2001 under a research contract, which ended in 2001, between Hexal AG, (Industriestraβe 25, 83607 Holzkirchen, Germany) and the Medical University of South Carolina to develop bacteriophage-based antimicrobial therapeutics. No products or patents based on this work or any bacteriophage therapeutic are being pursued by either Hexal AG or the Medical University of South Carolina. The author declares no non-financial competing interests.

## Authors' contributions

LK conceived of and designed the study, carried out the experiments, analyzed the data and prepared the manuscript.
